# Severe and uncontrolled adult asthma is associated with vitamin D insufficiency and deficiency

**DOI:** 10.1186/1465-9921-14-25

**Published:** 2013-02-22

**Authors:** Stephanie Korn, Marisa Hübner, Matthias Jung, Maria Blettner, Roland Buhl

**Affiliations:** 1 Pulmonary Department, Mainz University Hospital, Langenbeckstrasse 1, 55131, Mainz, Germany; 2 Institute of Medical Biostatistics, Epidemiology, and Informatics, Mainz University Hospital, Langenbeckstrasse 1, 55131, Mainz, Germany

**Keywords:** Asthma, BMI, Corticosteroids, Eosinophils, Vitamin D

## Abstract

**Background:**

Vitamin D has effects on the innate and adaptive immune system. In asthmatic children low vitamin D levels are associated with poor asthma control, reduced lung function, increased medication intake, and exacerbations. Little is known about vitamin D in adult asthma patients or its association with asthma severity and control.

**Methods:**

Clinical parameters of asthma control and 25-hydroxyvitamin D (25(OH)D) serum concentrations were evaluated in 280 adult asthma patients (mean ± SD: 45.0 ± 13.8 yrs., 40% male, FEV1 74.9 ± 23.4%, 55% severe, 51% uncontrolled).

**Results:**

25(OH)D concentrations in adult asthmatics were low (25.6 ±11.8 ng/ml) and vitamin D insufficiency or deficiency (vitamin D <30 ng/ml) was common (67%). 25(OH)D levels were related to asthma severity (intermittent: 31.1 ± 13.0 ng/ml, mild: 27.3 ± 11.9 ng/ml, moderate: 26.5 ± 12.0 ng/ml, severe: 24.0 ± 11.8 ng/ml, p = 0.046) and control (controlled: 29.5 ± 12.5 ng/ml, partly controlled 25.9 ± 10.8 ng/ml, uncontrolled: 24.2 ± 11.8 ng/ml, p = 0.030). The frequency of vitamin D insufficiency or deficiency was significantly higher in patients with severe or uncontrolled asthma and was associated with a lower FEV1 (vitamin D <30 vs. ≥30 ng/ml 2.3 ± 0.9 L vs. 2.7 ± 1.0 L, p = 0.006), higher levels of exhaled NO (45 ± 46 ppb vs. 31 ± 37 ppb, p = 0.023), a higher BMI (28.3 ± 6.2 vs. 25.1 ± 3.9, p < 0.001), and sputum eosinophilia (5.1 ± 11.8% vs. 0.5 ± 1.0%, p = 0.005). The use of oral corticosteroids or sputum eosinophilia was associated with a 20% or 40% higher risk of vitamin D insufficiency or deficiency.

**Conclusions:**

25(OH)D levels below 30 ng/ml are common in adult asthma and most pronounced in patients with severe and/or uncontrolled asthma, supporting the hypothesis that improving suboptimal vitamin D status might be effective in prevention and treatment of asthma.

## Background

Asthma represents one of the most common chronic diseases and is a major public health problem worldwide [1]. In the majority of patients control of asthma as defined by guidelines can be achieved with long-term maintenance medications [1]. However, a substantial proportion of patients do not achieve optimal asthma control despite even high dose treatment. In particular inadequately controlled patients with severe persistent asthma are at high risk of severe exacerbations and asthma-related mortality. These patients represent the greatest unmet medical need among the asthmatic population today.

Vitamin D insufficiency is increasingly recognized in the general population, and has been largely attributed to dietary, lifestyle and behavioral changes [2,3]. While its musculoskeletal consequences are well established, a new hypothesis links asthma to subnormal vitamin D levels [3-6]. Vitamin D has several effects on the innate and adaptive immune systems that might be relevant in the primary prevention of asthma, in the protection against or reduction of asthma morbidity, and in the modulation of the severity of asthma exacerbations [3,7,8]. Cross-sectional data indicate that low 25(OH)D levels in patients with mild to moderate asthma are correlated with poor asthma control, reduced lung function, reduced glucocorticoid response, more frequent exacerbations, and consequent increased steroid use [7,9-14]. However, there is insufficient evidence to support a causal association between vitamin D status and asthma per se. More so, there are very limited data in adult asthma patients addressing the impact of vitamin D status on disease control and severity. Therefore, the aim of this study was to prospectively investigate the prevalence of vitamin D insufficiency and deficiency in adult patients with asthma and its potential relationship with parameters of asthma severity and control, with a particular focus on patients with severe and uncontrolled disease.

## Methods

The study was approved by the local ethics committees (Ethikkommission der Landesärztekammer Rheinland-Pfalz, Mainz, Germany) and by the Institutional Review Board. The study was conducted in accordance with the ethical principles embodied in the Declaration of Helsinki and local applicable laws and regulations. All patients provided written informed consent prior to taking part in the study.

### Subjects

25-Hydroxyvitamin D3 (hereafter referred to as 25(OH)D) and clinical parameters of asthma severity and control were measured in 280 consecutive adult patients (≥ 18 years, all caucasians) with a previous physician diagnosis of asthma (Table 1) and 40 healthy volunteers (employees of Mainz University Hospital) between September 2008 and November 2011.

**Table 1:** Characteristics of patients with asthma and healthy volunteers

	**Asthma (n = 280)**	**Healthy controls (n = 40)**	**p-value**
**Age (years)**	45.0 ± 13.8	37.7 ± 13.5	0.002
**Gender (male), n (%)**	111 (40)	20 (50)	0.232
**Allergy, n (%)**	236 (84)	22 (55)	<0.001
**FEV1 (L)**	2.5 ± 0.9	3.8 ± 1.0	<0.001
**FEV1 (% of pred.)**	74.9 ± 23.4	103.6 ± 13.3	<0.001
**Exhaled NO (ppb)**	40.6 ± 41.5	19.6 ± 9.5	<0.001
**ICS use, n (%)**	202 (82)	-	-
**OCS use, n (%)**	75 (28)	-	-
**Asthma severity, n (%)**		-	
• **Intermittent**	• 18 (6.4)		
• **Mild**	• 54 (19.3)		
• **Moderate**	• 53 (18.9)		
• **Severe**	• 155 (55.4)		
**Asthma control, n (%)**		-	
• **Controlled**	• 44 (15.7)		
• **Partly controlled**	• 92 (32.9)		
• **Uncontrolled**	• 144 (51.4)		

ICS: inhaled corticosteroids, OCS: oral corticosteroids.

Displayed are means ± SD for continuous endpoints and frequencies for categorical endpoints.

Blood samples for 25(OH)D measurement were always taken in the morning between 8 and 11 am. Medical history, lung function tests, measurement of exhaled nitric oxide concentrations (FeNO) and sputum induction were performed on the same day. Serum levels of 25(OH)D were quantified by a radioimmunoassay (Cobra Quantum, Packard, MN, USA) and categorized into sufficient (≥ 30 ng/ml), insufficient (20 – < 30 ng/ml) or deficient (<20 ng/ml) based on previous recommendations [2,15,16]. To simplify matters, patients were categorized into 25(OH)D sufficient (≥30 ng/ml) or insufficient (0 - <30 ng/ml) unless specified otherwise. Serum levels of 25-hydroxyvitamin D3 are considered the best circulating biomarker of vitamin D metabolic status and reflect contributions from all sources of vitamin D (i.e., diet and sun exposure) [17,18]. Very few patients (n = 5) on nutritional supplements with a potential effect on 25(OH)D serum concentrations were excluded. In addition, interleukin-10 (IL-10) was measured in serum in all patients with asthma using an interleukin-10 ELISA (ImmunoTools GmbH, Friesoythe, Germany).

Asthma diagnosis was confirmed by either pre- and post-bronchodilator spirometry or methacholine bronchial challenge test. Classification of asthma severity was based on symptoms and asthma therapy as recommended [19]. Asthma control was assessed according to the criteria of the Global Initiative for Asthma using a categorical scale to identify controlled, partly controlled or uncontrolled asthma [19].

Allergy was defined as a positive skin prick test or allergen-specific IgE (ImmunoCAP, Phadia, Uppsala, Sweden) in combination with allergic symptoms. Lung function and FeNO were assessed following ATS/ERS guidelines. Regular medications were recorded in all patients, and asthma severity and control was defined based on symptom load and treatment intensity [20]. In a subset of patients sputum induction was performed and samples processed following established standards [21]. Eosinophils were counted and patients were categorized as eosinophilic (eosinophil count ≥ 3%) or non-eosinophilic.

The study was approved by the Institutional Review Board and was conducted in accordance with the ethical principles embodied in the Declaration of Helsinki and local applicable laws and regulations. All patients provided written informed consent prior to taking part in the study.

### Statistical analysis

The analyses evaluated the relationship between serum levels of 25(OH)D and GINA-defined asthma control, asthma severity and clinical and functional characteristics of asthma. Descriptive statistics were used to summarize patient characteristics relative to the four severity and three GINA asthma control categories. The analyses were further based on a categorization of patients into 25(OH)D sufficient (≥30 ng/ml) or insufficient (0 - < 30 ng/ml). Data description was primarily based on means and standard deviations (SD, normal data) for continuous endpoints, and on frequencies for categorical endpoints. Unadjusted comparisons between patients and control group were made using the t-test or Mann-Whitney U test for continuous endpoints and the Chi-Square test for categorical endpoints. To express the risk of vitamin D insufficiency relative risks, odds ratios (OR) and 95% confidence intervals (CI) were used. To determine the association between 25(OH)D levels or vitamin D insufficiency and disease severity, control and physiologic or inflammatory markers regression analyses were used with adjusted models for the potentially confounding effects of age, sex, BMI, and seasonality (4 categories). Correlations were assessed using the Pearson correlation (normal data) or the Spearman's rank correlation (skewed data). P values < 0.05 indicate local statistical significance and will be presented without adjustment for multiple testing. Data analysis was performed using SPSS® software (version11.5).

## Results

### Subjects

A total of 280 adult patients with asthma and 40 healthy volunteers as a control group were enrolled. 155 patients had severe asthma (55%) and 144 were uncontrolled (51%; Table 1). In 76 patients sputum induction was performed with a mean of 3.9 (±10.4)% eosinophils (mean ± SD) and 29% of patients were eosinophilic (≥3% eosinophils in sputum).

### 25(OH)D levels, asthma severity and asthma control

**Figure 1:**
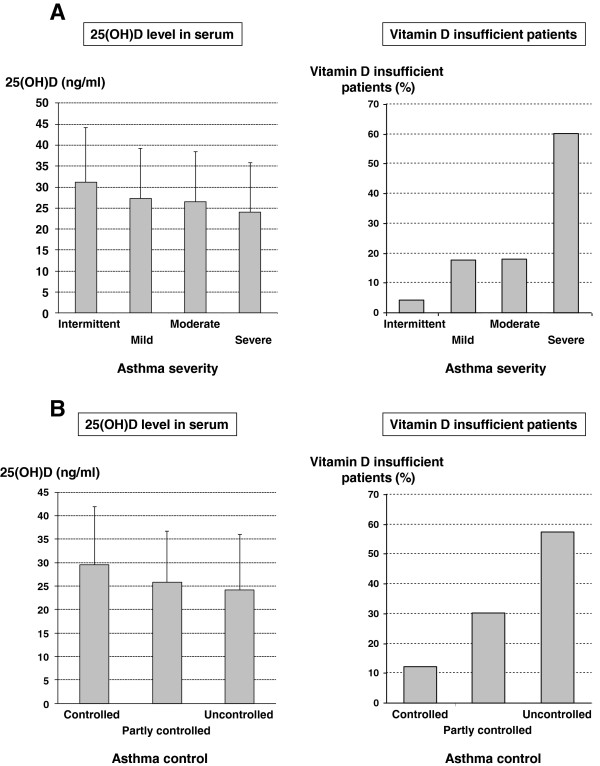
**A: 25(OH)D level in serum, vitamin D insufficiency and asthma severity.** Left: 25(OH)D level in serum (mean ± SD) for different asthma severity grades. Right: Percentage of vitamin D insufficient patients stratified by asthma severity. **B**: 25(OH)D level in serum, vitamin D insufficiency and asthma control. Left: 25(OH)D level in serum (mean ± SD) stratified by asthma control. Right: Percentage of vitamin D insufficient patients stratified by asthma control.

Mean 25(OH)D concentrations were 25.6 (±11.8) ng/ml in asthmatics and 26.2 (±16.8) ng/ml in healthy volunteers (p = 0.778). In the asthma population 35.4% of patients had normal 25(OH)D levels, 31.8% were vitamin D insufficient (20–29 ng/ml) and 32.9% were vitamin D deficient (0–19 ng/ml). Serum levels of 25(OH)D were significantly related to asthma severity (intermittent: 31.1 ± 13.0 ng/ml, mild: 27.3 ± 11.9 ng/ml, moderate: 26.5 ± 12.0 ng/ml, severe: 24.0 ± 11.8 ng/ml, p = 0.046) and asthma control (controlled: 29.5 ± 12.5 ng/ml, partly controlled 25.9 ± 10.8 ng/ml, uncontrolled: 24.2 ± 12.1 ng/ml, p = 0.030) (Figure 1). About 75% of patients with severe or uncontrolled asthma were vitamin D insufficient as defined by a level of 30 ng/ml or less. Patients with severe and uncontrolled asthma had the lowest 25(OH)D levels (23.7 ± 12.3 ng/ml) compared with patients with intermittent, mild or moderate and controlled or partly controlled asthma (27.1 ± 11.7 ng/ml, p = 0.014). Patients with severe or uncontrolled asthma had a 20% or 30% higher risk to be vitamin D insufficient compared with patients with intermittent, mild or moderate disease or with controlled or partly controlled asthma, respectively. The odds ratio for being vitamin D insufficient for patients with severe or uncontrolled asthma was 1.9 (95% CI 1.2–3.2) and 2.1 (1.3–3.5), respectively.

### 25(OH)D levels and clinical characteristics of asthma

**Figure 2:**
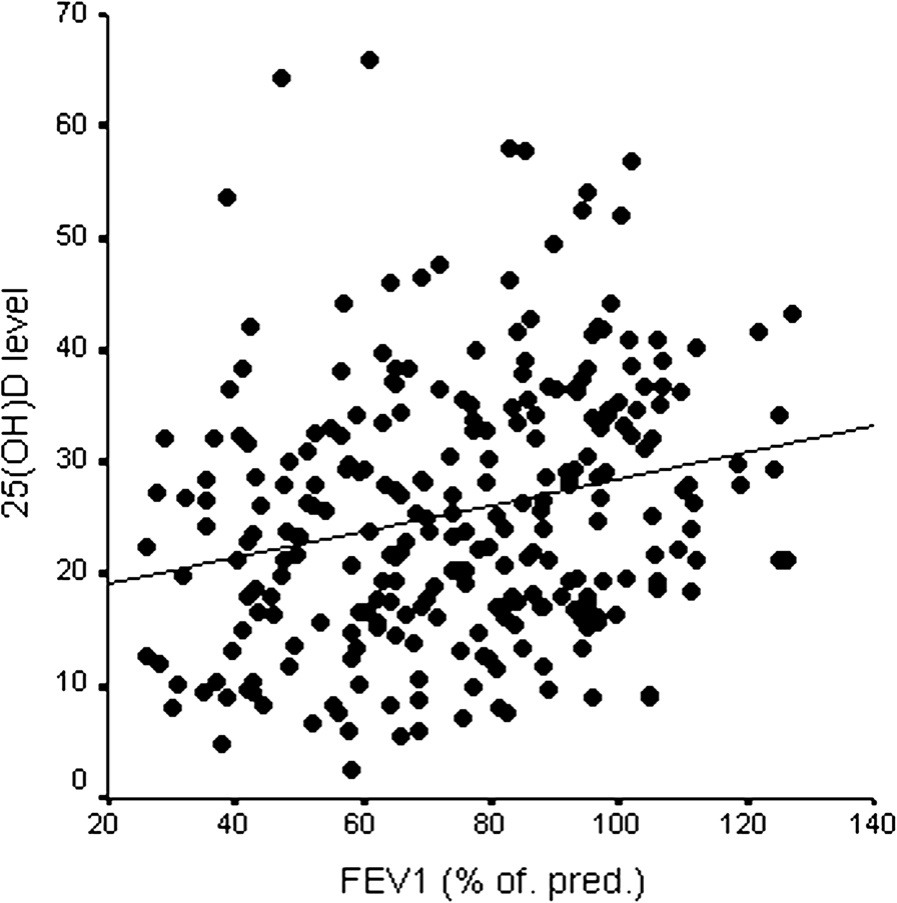
25(OH)D levels (ng/ml) and FEV1 (% of predicted), r = 0.235, p < 0.001.

In patients with asthma, 25(OH)D levels were positively correlated with FEV1 (r = 0.235, p < 0.001, Figure 2). Vitamin D insufficiency was associated with a lower FEV1 (vitamin D < 30 ng/ml vs. ≥ 30 ng/ml: 2.3 ± 0.9 L vs. 2.7 ± 1.0 L, p = 0.005; 71.1 ± 23.4% pred. vs. 81.4 ± 22.3%, p = 0.001), higher levels of exhaled NO (45 ± 46 ppb vs. 31 ± 27 ppb, p = 0.023) and sputum eosinophilia (5.1 ± 11.8% vs. 0.5 ± 1.0%, p = 0.005, Table 2). The difference in sputum eosinophils between vitamin D sufficient and vitamin D insufficient patients remains significant (p = 0.004) after adjustment for potential confounders (age, gender, BMI, season). Patients with a sputum eosinophil count ≥ 3% had a 40% higher risk of being vitamin D insufficient compared with patients without sputum eosinophilia, the odds ratio for vitamin D insufficiency in patients with sputum eosinophilia was 10.5 (CI 1.0–83.3). No association was observed between 25(OH)D and serum IgE levels (Table 2).

**Table 2:** Vitamin D insufficiency and clinical characteristics of asthma

	**25(OH)D** **<** **30 ng/ml**	**25(OH)D **≥ **30 ng/ml**	**p-value**
Allergy			
Yes	159 (67.4%)	77 (32.6%)	
No	28 (66.7%)	14 (33.3%)	0.359
**OCS use**			
**Yes**	**58 (77.3%)**	**17 (22.7%)**	
**No**	**130 (63.4%)**	**75 (36.6%)**	**0.031**
OCS dose (mg/d)	24 ± 29	16 ± 12	0.274
ICS use			
Yes	150 (68.2%)	70 (31.8%)	
No	38 (63.3%)	22 (36.7%)	0.536
ICS dose (μg/d)	1310 ± 815	1232 ± 916	0.547
Age (years)	44.5 ± 13.0	46.0 ± 15.4	0.400
**FEV1 (L)**	**2.3 ± 0.9**	**2.7 ± 1.0**	**0.003**
**FEV1 (%)**	**71.7 ± 23.4**	**81.4 ± 22.3**	**0.001**
Reversibility (%)	13.1 ± 22.7	12.2 ± 23.3	0.838
**Exhaled NO (ppb)**	**45 ± 46**	**31 ± 27**	**0.022**
Total IgE (IU/ml)	544 ± 1292	365 ± 689	0.230
Duration of disease (years)	18.6 ± 13.5	17.5 ± 15.5	0.539
**Sputum eosinophilia**			
**Yes**	**21 (95.5%)**	**1 (4.5%)**	
**No**	**36 (66.7%)**	**18 (33.3%)**	**0.008**
**Sputum eosinophils (%)**	**5.1 ± 11.9**	**0.5 ± 1.0**	**0.005**
**BMI (kg/m**^**2**^**)**	**28.3 ± 6.3**	**25.1 ± 3.9**	**<0.001**
IL-10 (ng/ml)	118.6 ± 122.6	114.2 ± 137.1	0.788

Displayed are means ± SD for continuous endpoints and frequencies for categorical endpoints.

Of the different therapies prescribed to asthma patients, the use of daily maintenance oral steroids was significantly associated with lower 25(OH)D levels (Table 2). Patients using oral corticosteroids had a 20% higher risk to be vitamin D insufficient than patients without maintenance oral corticosteroids (OR 2.0, CI 1.1–3.6). There was no association between the use and daily dose of ICS and 25(OH)D levels.

### 25(OH)D levels and body mass

In patients with asthma, 25(OH)D levels were inversely correlated with BMI (r = −0.278, p < 0.001). Vitamin D-insufficient patients had a significantly higher BMI (28.3 ± 6.2 vs. 25.1 ± 3.9, p < 0.001).

### 25(OH)D levels and IL-10

There was no correlation of 25(OH)D levels in serum and serum IL-10 concentrations (Table 2). IL-10 levels in patients with more severe disease and uncontrolled asthma did not differ from levels in patients with mild and moderate or controlled and partly controlled disease (113.5 ± 116.8 ng/ml vs. 120.2 ± 135.6, p = 0.658).

### 25(OH)D levels and seasonality

**Figure 3:**
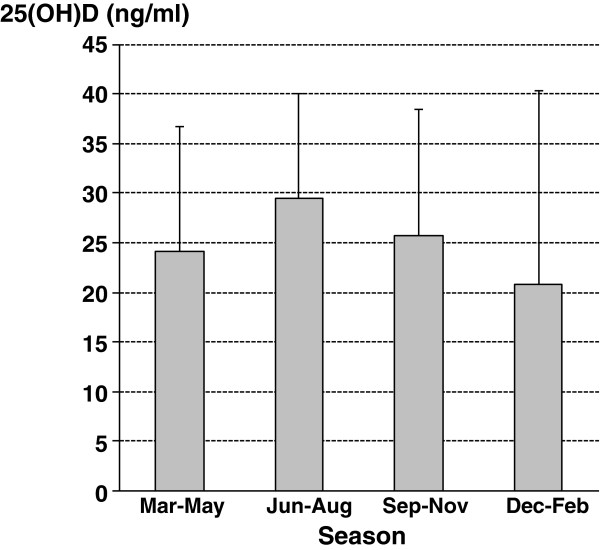
25(OH)D levels in serum during the seasons.

Blood was taken from March to May in 79 asthmatics, from June to August in 91 asthmatics, from September to November in 58 asthmatics and from December to February in 52 asthmatics. 25(OH)D concentrations varied by season with highest levels in summer and lowest levels in winter and spring (Figure 3). Over the collection period of 3 years there was a similar number of samples collected during the 4 seasons and asthma severity and asthma control grades were equally distributed over the seasons. Introducing season as an additional covariable did not change the results of the previous analyses.

## Discussion

The present cross-sectional study demonstrates in a large, well-characterized population of adult asthma patients of all degrees of severity and different grades of control over a period of 3 years a highly significant correlation between vitamin D status (as reflected by serum 25(OH)D concentrations) and measures of asthma severity and control. There is an inverse relationship between 25(OH)D and asthma severity and a positive relationship between 25(OH)D and both lung function and asthma control. Lower 25(OH)D levels are associated with worse lung function, higher levels of exhaled NO, sputum eosinophilia, oral corticosteroid use and higher BMI. The frequency of vitamin D insufficiency was highest in patients with severe, uncontrolled asthma, in particular in patients with a sputum eosinophil count ≥3% despite treatment with inhaled and oral corticosteroids. The findings of the present study confirm and extend in adult patients with various degrees of asthma severity reports in which vitamin D status is associated with asthma severity and control in children [7,9,22,23]. There is a paucity of data on the relation between vitamin D status and lung function in the general population, and our findings are in line with those from the large cross-sectional NHANES III study in the USA which reported a difference of more than 100 ml in FEV1 between those in the top and bottom quintiles for serum vitamin D concentrations [24]. Similarly, our results are well in line with a Chinese study demonstrating a positive association between vitamin D status and lung function [12].

Sun exposure is the main source of vitamin D in humans. Vitamin D skin metabolism is influenced by melanin content of the skin, by age, by factors affecting sun exposure, and by body fat. Dietary intake and supplements are a secondary source of vitamin D. Recent epidemiologic studies suggest that the prevalence of vitamin D insufficiency in the population is increasing; a phenomenon attributed at least in part to dietary and behavioral changes over the last decades [3,25]. Studies investigating the link between vitamin D insufficiency and asthma yielded conflicting results. As yet there is insufficient evidence of a causal association between vitamin D status and asthma per se or a major role of vitamin D status in asthma morbidity [26]. It is unclear if the association of 25(OH)D concentrations with asthma severity and control is a consequence of lifestyle and dietary changes due to asthma (e.g. less time spent outdoors, medication) or if asthma severity and control are negatively influenced by vitamin D insufficiency that occurred independently of asthma morbidity or asthma control. Ongoing clinical trials should be able to answer this question [3], even though the largest study includes patients with mild asthma, the population in which the association between vitamin D insufficiency and disease severity and control is least pronounced. Clearly, lower 25(OH)D levels in patients with more severe disease are no seasonal phenomenon. In the present study, the long study period of some 3 years and the expected pattern, higher 25(OH)D concentrations in summer and autumn compared with winter and spring in all patients irrespective of disease severity, exclude a seasonal bias.

The strong correlation between asthma severity as well as disease control (e.g. FEV1, FeNO, sputum eosinophils) and 25(OH)D concentrations suggests an impact of hormonal effects on the asthmatic inflammation or vice versa. Irrespective of the mechanisms underlying the association between vitamin D insufficiency and asthma several lines of evidence support a major role of vitamin D in asthma and the observed inverse correlation between vitamin D status and asthma severity and control. Airway epithelia contain high levels of the enzyme that converts circulating 25-OH-vitamin D3 to its active form, 1,25-OH-vitamin D3. The active form of vitamin D has local effects in response to respiratory infections and might dampen the inflammation that is the consequence of these infections [27]. Reduced vitamin D levels are associated with increased expression of TNF-alpha, suggesting that enhanced expression of this pro-inflammatory cytokine is a potential pathway by which reduced vitamin D levels could exert pro-inflammatory effects in asthma [28,29]. Vitamin D also has potentially beneficial effects on the adaptive immune system through its effects on T cells [30], promoting differentiation of naive T cells into IL-10–secreting regulatory T cells [31,32], and increasing serum levels of the immune-modulatory cytokines TGF-β and IL-10 [32,33]. Further, recent data suggest that vitamin D interacts with glucocorticoid signaling pathways in ways that are clinically relevant [34], and that vitamin D may potentially improve glucocorticoid responsiveness in severe asthmatics by up-regulation of IL-10 production from CD4+ cells [34]. This may be relevant even though in the present study no link between vitamin D status and IL-10 serum concentrations was observed. A small study in adults with mild or moderate persistent asthma demonstrated that lower serum vitamin D concentrations were associated with impaired lung function, increased airway hyperresponsiveness, and decreased in vitro corticosteroid response, with higher serum vitamin D concentrations associated with enhanced dexamethasone-induced expression of mitogen activated protein kinase phosphatase-1 by PBMCs in an apparently IL-10–independent fashion [11]. Finally, a study with bronchial biopsies demonstrated an inverse association of vitamin D levels and airway smooth muscle mass [22]. In vitro vitamin D influenced airway smooth muscle remodeling by exerting an inhibitory effect on passively sensitized airway smooth muscle growth and contractility [35].

Of particular relevance is the observed inverse relationship between BMI and 25(OH)D levels, a finding previously reported in adults without asthma [36]. Obesity has been demonstrated to increase asthma risk [37], and one of the most significant effects of obesity in asthma relates to its association with an impaired response to glucocorticosteroids [38,39]. Higher vitamin D levels in adults with asthma are not only correlated with improved lung function and reduced bronchial hyperresponsiveness, but also with an improved in vitro response to glucocorticosteroids [11]. The present findings suggest that reduced 25(OH)D levels in overweight and obese asthma patients may contribute to the reduced glucocorticosteroid response in this population.

With this as background, and irrespective of body weight it is tempting to speculate on a potential role of vitamin D supplementation in patients with suboptimal asthma control despite treatment with inhaled and systemic glucocorticosteroids, in particular in severe and / or uncontrolled asthma. Recent studies in children with asthma showed a significant inverse association between vitamin D levels and use of anti-inflammatory asthma medication (either ICS or leukotriene inhibitors) in the previous year, total IgE levels, and eosinophil counts [9,23]. Interestingly, in contrast to a similar study in childhood asthma [22] the present study demonstrated a strong association between vitamin D insufficiency and sputum eosinophilia despite treatment with inhaled and systemic corticosteroid treatment. This discrepancy is potentially due to the fact that these criteria define an asthma phenotype, eosinphilic asthma, that, if at all present, is not very prevalent in childhood asthma. This hypothesis is in line with the fact that eosinophilc asthma is characterized by late disease onset [40].

The present study has limitations: Even though there is a strong relation of asthma severity, asthma control and 25(OH)D level, the design of the study does not allow conclusions about cause or effect of vitamin D insufficiency. In contrast to controlled trials the present study was not based on a study protocol with in- and exclusion criteria or matched controls. As a consequence the study population was not homogeneous. The patients included in this study are typical for a large asthma referral center and may therefore not be representative of the overall population of patients with asthma but reflect real-life conditions. However, given the large overall study population it is unlikely that the results change if more patients with milder disease were included. More so, patients with more severe disease are potentially the population in which vitamin D insufficiency is most relevant. Another controversial issue is that regardless of the threshold used, vitamin D insufficiency has increased in industrialized countries over the last decades due to changes in behavior and diet [25], i.e. the observed low levels may be just coincidental. This fact questions the relevance of low vitamin D serum levels.

In summary, the present study demonstrates for the first time that 25(OH)D levels are associated with clinical parameters of asthma severity and asthma control in adult patients with asthma. Frequency of vitamin D insufficiency is highest in patients with severe and uncontrolled asthma. This is even more relevant given that the risk of vitamin D insufficiency is significantly increased in patients on oral corticosteroids or with eosinophilic disease.

## Competing interests

No author has any competing interests.

## Authors’ contributions

SK and RB made substantial contributions to concept and design of study. SK, MH and MJ performed study visits and collected data. MB, SK and RB contributed to the analysis and interpretation of data. All authors critically reviewed the report and approved the final version.

## References

[B1] Global Initiative for Asthma GINA Report, Global Strategy for Asthma Management and Prevention - revised 2010 2010 Available at: [http://www.ginasthma.org]

[B2] Holick MF Vitamin D deficiency N Engl J Med 2007 357 266 281 10.1056/NEJMra070553 17634462

[B3] Paul G Brehm JM Alcorn JF Holguin F Aujla SJ Celedon JC Vitamin D and asthma Am J Respir Crit Care Med 2012 185 124 132 10.1164/rccm.201108-1502CI 22016447 PMC3297088

[B4] Camargo CA Jr Rifas-Shiman SL Litonjua AA Rich-Edwards JW Weiss ST Gold DR Kleinman K Gillman MW Maternal intake of vitamin D during pregnancy and risk of recurrent wheeze in children at 3 y of age Am J Clin Nutr 2007 85 788 795 17344501 10.1093/ajcn/85.3.788 PMC4406411

[B5] Litonjua AA Weiss ST Is vitamin D deficiency to blame for the asthma epidemic? J Allergy Clin Immunol 2007 120 1031 1035 10.1016/j.jaci.2007.08.028 17919705

[B6] Ginde AA Sutherland ER Vitamin D in asthma: Panacea or true promise? J Allergy Clin Immunol 2010 126 59 60 10.1016/j.jaci.2010.05.030 20620566

[B7] Brehm JM Schuemann B Fuhlbrigge AL Hollis BW Strunk RC Zeiger RS Weiss ST Litonjua AA Serum vitamin D levels and severe asthma exacerbations in the Childhood Asthma Management Program study J Allergy Clin Immunol 2010 126 1 52 8.e5 Epub 2010 Jun 9 10.1016/j.jaci.2010.03.043 20538327 PMC2902692

[B8] Goleva E Searing DA Jackson LP Richers BN Leung DY Steroid requirements and immune associations with vitamin D are stronger in children than adults with asthma J Allergy Clin Immunol 2012 129 1243 1251 10.1016/j.jaci.2012.01.044 22330698 PMC3340468

[B9] Brehm JM Celedon JC Soto-Quiros ME Avila L Hunninghake GM Forno E Laskey D Sylvia JS Hollis BW Weiss ST Litonjua AA Serum vitamin D levels and markers of severity of childhood asthma in Costa Rica Am J Respir Crit Care Med 2009 179 765 771 10.1164/rccm.200808-1361OC 19179486 PMC2675563

[B10] Chinellato I Piazza M Sandri M Peroni D Piacentini G Boner AL Vitamin D serum levels and markers of asthma control in Italian children J Pediatr 2011 158 437 441 10.1016/j.jpeds.2010.08.043 20870246

[B11] Sutherland ER Goleva E Jackson LP Stevens AD Leung DY Vitamin D levels, lung function, and steroid response in adult asthma Am J Respir Crit Care Med 2010 181 699 704 10.1164/rccm.200911-1710OC 20075384 PMC2868500

[B12] Li F Peng M Jiang L Sun Q Zhang K Lian F Litonjua AA Gao J Gao X Vitamin D deficiency is associated with decreased lung function in Chinese adults with asthma Respiration 2011 81 469 475 10.1159/000322008 21124013 PMC3124457

[B13] Brehm JM Costa-Perez E Klei L Roeder K Barmada M Boutaoui N Forno E Kelly R Paul K Sylvia J Litonjua AA Cabana M Alvarez M Colon-Semidey A Canino G Celedon JC Vitamin D Insufficiency and Severe Asthma Exacerbations in Puerto Rican Children Am J Respir Crit Care Med 2012 186 140 146 10.1164/rccm.201203-0431OC 22652028 PMC3406083

[B14] Wu AC Tantisira K Li L Fuhlbrigge AL Weiss ST Litonjua A The Effect of Vitamin D and Inhaled Corticosteroid Treatment on Lung Function in Children Am J Respir Crit Care Med 2012 186 6 508 13 Epub 2012 Jul 12 10.1164/rccm.201202-0351OC 22798322 PMC3480528

[B15] Holick MF Binkley NC Bischoff-Ferrari HA Gordon CM Hanley DA Heaney RP Murad MH Weaver CM Evaluation, treatment, and prevention of vitamin D deficiency: an Endocrine Society clinical practice guideline J Clin Endocrinol Metab 2011 96 1911 1930 10.1210/jc.2011-0385 21646368

[B16] Vieth R Bischoff-Ferrari H Boucher BJ Dawson-Hughes B Garland CF Heaney RP Holick MF Hollis BW Lamberg-Allardt C McGrath JJ Norman AW Scragg R Whiting SJ Willett WC Zittermann A The urgent need to recommend an intake of vitamin D that is effective Am J Clin Nutr 2007 85 649 650 17344484 10.1093/ajcn/85.3.649

[B17] Hollis BW Circulating 25-hydroxyvitamin D levels indicative of vitamin D sufficiency: implications for establishing a new effective dietary intake recommendation for vitamin D J Nutr 2005 135 317 322 15671234 10.1093/jn/135.2.317

[B18] Hollis BW Wagner CL Normal serum vitamin D levels N Engl J Med 2005 352 515 516 15689596 10.1056/NEJM200502033520521

[B19] Global Initiative for Asthma GINA Report, Global Strategy for Asthma Management and Prevention - updated 2008 2008 Available at: [http://www.ginasthma.org]

[B20] Global Initiative for Asthma GINA Report, Global Strategy for Asthma Management and Prevention - updated 2006 2006 Available at: [http://www.ginasthma.org]

[B21] Beeh KM Beier J Kornmann O Mander A Buhl R Long-term repeatability of induced sputum cells and inflammatory markers in stable, moderately severe COPD Chest 2003 123 778 783 10.1378/chest.123.3.778 12628878

[B22] Gupta A Sjoukes A Richards D Banya W Hawrylowicz C Bush A Saglani S Relationship Between Serum Vitamin D, Disease Severity and Airway Remodeling in Children with Asthma Am J Respir Crit Care Med 2011 184 12 1342 9 Epub 2011 Sep 8 10.1164/rccm.201107-1239OC 21908411 PMC3471128

[B23] Searing DA Zhang Y Murphy JR Hauk PJ Goleva E Leung DY Decreased serum vitamin D levels in children with asthma are associated with increased corticosteroid use J Allergy Clin Immunol 2010 125 995 1000 10.1016/j.jaci.2010.03.008 20381849 PMC2866800

[B24] Black PN Scragg R Relationship between serum 25-hydroxyvitamin d and pulmonary function in the third national health and nutrition examination survey Chest 2005 128 3792 3798 10.1378/chest.128.6.3792 16354847

[B25] Ginde AA Liu MC Camargo CA Jr Demographic differences and trends of vitamin D insufficiency in the US population, 1988–2004 Arch Intern Med 2009 169 626 632 10.1001/archinternmed.2008.604 19307527 PMC3447083

[B26] Devereux G Wilson A Avenell A McNeill G Fraser WD A case-control study of vitamin D status and asthma in adults Allergy 2010 65 666 667 10.1111/j.1398-9995.2009.02220.x 19845573

[B27] Hansdottir S Monick MM Hinde SL Lovan N Look DC Hunninghake GW Respiratory epithelial cells convert inactive vitamin D to its active form: potential effects on host defense J Immunol 2008 181 7090 7099 18981129 10.4049/jimmunol.181.10.7090 PMC2596683

[B28] Berry MA Hargadon B Shelley M Parker D Shaw DE Green RH Bradding P Brightling CE Wardlaw AJ Pavord ID Evidence of a role of tumor necrosis factor alpha in refractory asthma N Engl J Med 2006 354 697 708 10.1056/NEJMoa050580 16481637

[B29] Mora JR Iwata M von Andrian UH Vitamin effects on the immune system: vitamins A and D take centre stage Nat Rev Immunol 2008 8 685 698 10.1038/nri2378 19172691 PMC2906676

[B30] Hawrylowicz CM O'Garra A Potential role of interleukin-10-secreting regulatory T cells in allergy and asthma Nat Rev Immunol 2005 5 271 283 10.1038/nri1589 15775993

[B31] Barrat FJ Cua DJ Boonstra A Richards DF Crain C Savelkoul HF de Waal-Malefyt R Coffman RL Hawrylowicz CM O'Garra A In vitro generation of interleukin 10-producing regulatory CD4(+) T cells is induced by immunosuppressive drugs and inhibited by T helper type 1 (Th1)- and Th2-inducing cytokines J Exp Med 2002 195 603 616 10.1084/jem.20011629 11877483 PMC2193760

[B32] Urry Z Xystrakis E Richards DF McDonald J Sattar Z Cousins DJ Corrigan CJ Hickman E Brown Z Hawrylowicz CM Ligation of TLR9 induced on human IL-10-secreting Tregs by 1alpha,25-dihydroxyvitamin D3 abrogates regulatory function J Clin Invest 2009 119 387 398 19139565 10.1172/JCI32354 PMC2631286

[B33] Mahon BD Gordon SA Cruz J Cosman F Cantorna MT Cytokine profile in patients with multiple sclerosis following vitamin D supplementation J Neuroimmunol 2003 134 128 132 10.1016/S0165-5728(02)00396-X 12507780

[B34] Xystrakis E Kusumakar S Boswell S Peek E Urry Z Richards DF Adikibi T Pridgeon C Dallman M Loke TK Robinson DS Barrat FJ O'Garra A Lavender P Lee TH Corrigan C Hawrylowicz CM Reversing the defective induction of IL-10-secreting regulatory T cells in glucocorticoid-resistant asthma patients J Clin Invest 2006 116 146 155 16341266 10.1172/JCI21759 PMC1307558

[B35] Damera G Fogle HW Lim P Goncharova EA Zhao H Banerjee A Tliba O Krymskaya VP Panettieri RA Jr Vitamin D inhibits growth of human airway smooth muscle cells through growth factor-induced phosphorylation of retinoblastoma protein and checkpoint kinase 1 Br J Pharmacol 2009 158 1429 1441 10.1111/j.1476-5381.2009.00428.x 19814732 PMC2795210

[B36] Parikh SJ Edelman M Uwaifo GI Freedman RJ Semega-Janneh M Reynolds J Yanovski JA The relationship between obesity and serum 1,25-dihydroxy vitamin D concentrations in healthy adults J Clin Endocrinol Metab 2004 89 1196 1199 10.1210/jc.2003-031398 15001609

[B37] Beuther DA Sutherland ER Overweight, obesity, and incident asthma: a meta-analysis of prospective epidemiologic studies Am J Respir Crit Care Med 2007 175 661 666 10.1164/rccm.200611-1717OC 17234901 PMC1899288

[B38] Sutherland ER Lehman EB Teodorescu M Wechsler ME Body mass index and phenotype in subjects with mild-to-moderate persistent asthma J Allergy Clin Immunol 2009 123 1328 1334 10.1016/j.jaci.2009.04.005 19501235 PMC2743451

[B39] Haldar P Pavord ID Shaw DE Berry MA Thomas M Brightling CE Wardlaw AJ Green RH Cluster analysis and clinical asthma phenotypes Am J Respir Crit Care Med 2008 178 218 224 10.1164/rccm.200711-1754OC 18480428 PMC3992366

[B40] Haldar P Brightling CE Hargadon B Gupta S Monteiro W Sousa A Marshall RP Bradding P Green RH Wardlaw AJ Pavord ID Mepolizumab and exacerbations of refractory eosinophilic asthma N Engl J Med 2009 360 973 984 10.1056/NEJMoa0808991 19264686 PMC3992367

